# The gut microbiome: what the oncologist ought to know

**DOI:** 10.1038/s41416-021-01467-x

**Published:** 2021-07-14

**Authors:** K. A. Lee, M. K. Luong, H. Shaw, P. Nathan, V. Bataille, T. D. Spector

**Affiliations:** 1 Department of Twin Research and Genetic Epidemiology, King’s College London, London, UK; 2 Department of Medical Oncology, Mount Vernon Hospital, Northwood, UK; 3 Department of Medical Oncology, The Royal Marsden, London, UK; 4 Department of Medical Oncology, Guy’s & St Thomas Hospital, London, UK; 5 Early Phase Trial Unit, Department of Medical Oncology, University College London Hospital, London, UK; 6 Department of Dermatology, Mount Vernon Hospital, Northwood, UK

**Keywords:** Cancer microenvironment, Microbiome

## Abstract

The gut microbiome (GM) has been implicated in a vast number of human pathologies and has become a focus of oncology research over the past 5 years. The normal gut microbiota imparts specific function in host nutrient metabolism, xenobiotic and drug metabolism, maintenance of structural integrity of the gut mucosal barrier, immunomodulation and protection against pathogens. Strong evidence is emerging to support the effects of the GM on the development of some malignancies but also on responses to cancer therapies, most notably, immune checkpoint inhibition. Tools for manipulating the GM including dietary modification, probiotics and faecal microbiota transfer (FMT) are in development. Current understandings of the many complex interrelationships between the GM, cancer, the immune system, nutrition and medication are ultimately based on a combination of short‐term clinical trials and observational studies, paired with an ever-evolving understanding of cancer biology. The next generation of personalised cancer therapies focusses on molecular and phenotypic heterogeneity, tumour evolution and immune status; it is distinctly possible that the GM will become an increasingly central focus amongst them. The aim of this review is to provide clinicians with an overview of microbiome science and our current understanding of the role the GM plays in cancer.

## The gut microbiome

**Fig. 1:**
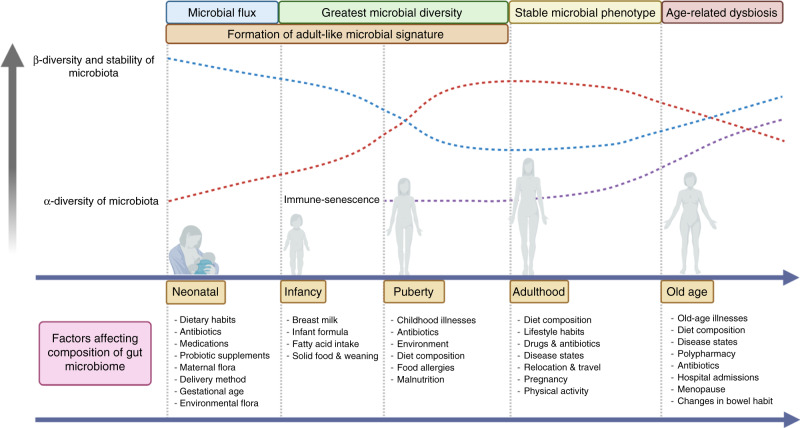
Development of the gut microbiome. This figure demonstrates the development of the gut microbiome from birth to old age in relation to α-diversity (diversity of species within the same individual), β-diversity (inter-individual species diversity) diversity and the relationship between immunity and senescence, as well as external factors that may affect the GM composition. Created with BioRender.com.

The gut microbiome (GM) refers to the genetic makeup of all microbes that exist within the human gastrointestinal tract, including bacteria, viruses, yeast, protozoa, fungi and archaea. The GM contains ~100 trillion micro-organisms, which encode over three million genes producing thousands of metabolites, which replace or modulate many of the functions of the human host [1]. Constituents of the GM have been shown to interact with one another and the host immune system in ways that influence physiological homeostasis and the development of disease. The normal human GM comprises two major phyla, namely *Bacteroidetes and Firmicutes*. Evidence of early microbial contact suggests that human intestinal microbiota is seeded in utero. Maternal microbiota forms the first microbial inoculum, and from birth, diversity increases and converges toward an adult-like microbiota by the end of the first 3–5 years of life. Various factors shape this colonisation, including mode of delivery, maternal and infant perinatal antibiotic exposure, feeding methods and dietary factors, amongst others (Fig. 1). Once established, the composition of the gut microbiota is relatively stable throughout the adult life but can be altered as a result of bacterial infections, antibiotic treatment, smoking, disease states, medical and surgical interventions and long-term dietary changes [2].

The GM has been referred to as “the last undiscovered human organ”; microbes within the human gut have significant effects on human health and immune function due to their proximity to the immune environment within the gastrointestinal tract. Complex interactions allow for the oral tolerance of commensal bacteria and food antigens, along with enabling the immune system to recognise and attack opportunistic bacteria. In addition to influencing localised immune responses, the microbiota also has broader effects contributing to innate and adaptive immunity by modulating the regulatory phenotype of gastrointestinal dendritic cells [3]. This concept is supported in animal models: germ-free mice lacking intestinal microbiota are noted to have severe defects in immunity, with an absent mucous layer, altered IgA secretion and reduced size and functionality of Peyer’s patches and draining mesenteric lymph nodes [4].

Lower gut bacterial diversity has been reproducibly observed in patients with a wide range of conditions, including obesity, cardiovascular disease, autoimmune disease and neurological disorders, along with vaccine responses, suggesting a direct relationship between GM diversity and functional outcomes [5–9]. Disruption of the delicate balance of commensal bacteria is seen in the setting of what is often referred to as *dysbiosis*, a somewhat ill-defined state characterised by a less diverse and less stable microbiota, with potential enrichment of opportunistic pathogenic bacteria [10]. Such an imbalance can lead to impaired local, locoregional and systemic immune responses through the breakdown of mucosal barriers, alterations in cytokine signalling, inhibition of probiotic commensal bacterial colonisation and proliferation of enteropathogens. This results in the translocation of gut bacteria into the mesenteric lymph nodes and peripheral circulation, resulting in the activation of Th17 and effector T cells promoting neutrophil infiltration, and activation of an inflammatory phenotype both locally and systemically [11].

## Sampling, sequencing and analysis

For pragmatic reasons, faecal specimens are frequently used as proxies for gut microbiota, as they can be collected non-invasively and repeatedly, and are the preferred method in most GM studies. However, there is increasing evidence to suggest that there may be significant variations in microbial composition between the gut mucosa and faeces [12]. Whilst deemed to be a substitute for gastrointestinal lumen contents, faecal contents may not accurately reflect the complex interactions that occur directly at the gut mucosal surface. In fact, it has been shown that faecal and mucosal-associated microbiotas are two distinct populations [13]. Furthermore, the faecal microbiota is not distributed equally within faeces and has its own unique biostructure [14].

Ideally, microbial genetic sequencing and analysis should be performed on fresh, uncontaminated faecal specimens. However, an ongoing challenge remains in that human faecal sampling generally cannot be produced on request, and patients would usually collect samples within their own homes, before being transported to a laboratory. Therefore, in order to maintain microbial DNA integrity, specimens should be immediately frozen at −80 °C without preservatives [15]. When optimal conditions for immediate storage of specimens at ultra-low temperatures cannot be met, storage and transportation at 4 °C can minimise further changes to the microbial composition [16]. Alternative strategies such as RNA*later*® and DNA/RNA Shield™ aim to overcome this limitation by utilising preservation media to stabilise cellular RNA and DNA by the inactivation of nucleases, which allows for samples to be stored at ambient temperatures from the time of collection, and eliminates the need for samples to be rapidly transferred to low-temperature environments [17]. The degree of variability in faecal sampling, therefore, demands the establishment of validated collection procedures to minimise any potential systemic bias that may be introduced in pre-processing steps [18]. Other GM sampling methods have been described and reviewed by Tang et al. [19].

Within the past 15 years, advancements in genomic sequencing techniques have accelerated our understanding of the GM. Researchers can now sequence entire genomes of microbial communities with shotgun metagenomics, providing a higher taxonomic resolution and the ability to extract the functional gene content of each genome, compared to 16S ribosomal RNA gene sequencing (phylogenomics) [20]. Analysis of metagenomic data together with the identification of specific metabolites could be used to develop predictive microbiome signatures, potentiating novel therapeutic interventions [21]. However, data obtained from sequencing is often voluminous, fragmented, noisy and contaminated. Bioinformatics analysis allows us to utilise computational methods to clean and analyse large volumes of biological data, with the subsequent identification of bacterial taxa. Microbial analysis may also reveal distinct ecosystems such as α-diversity (diversity of species within the same individual), β-diversity (inter-individual species diversity) and relative abundance. Given the complexity and volume of raw data, metagenomic workflows are typically employed to ensure quality control, quality assessments and accurate taxonomic characterisation. Shotgun metagenomics, however, is limited by a high noise-to-signal ratio generated between the host and bacteria. Alternative techniques aim to address this; for example, the development of high-throughput culture techniques such as culturomics, combined with mass spectrometric microbial identification techniques such as matrix-assisted laser desorption/ionisation time-of-flight [22], which have significantly expanded our knowledge of beneficial and fastidious bacterial gut strains and species, with hundreds of new taxa having been identified in recent years [23].

## The GM and colorectal cancer

**Fig. 2:**
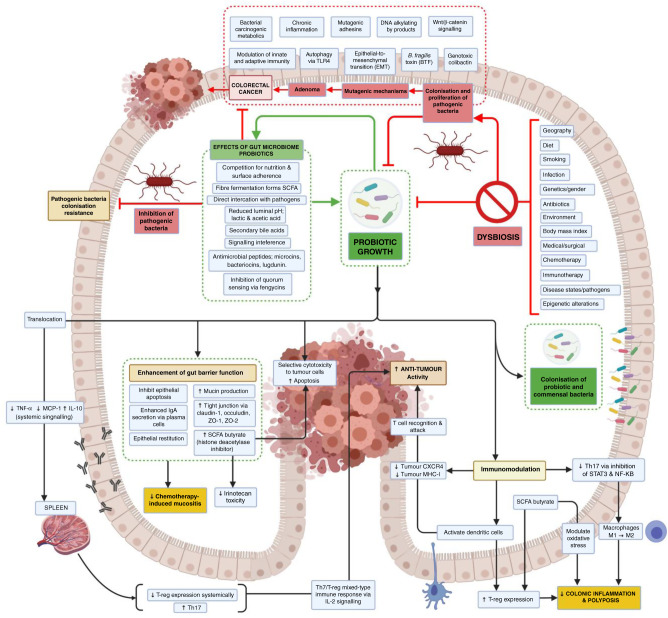
Summary of the putative mechanisms of therapeutic microbiota on gut microbiome homeostasis and colorectal cancer carcinogenesis by immune-mediated and non-immune-mediated mechanisms. Therapeutic microbiota may potential CRC prevention through (1) enhancement of gut barrier function, (2) immunomodulation (activation of DCs, macrophages, tumour CXCR4 and MHC-1, systemic Th7/T-reg immune response) and modulation of oxidative stress to reduce colonic inflammation and increase immune-mediated anti-tumour activity, (3) promotion of an advantageous gut microenvironment that inhibits pathogenic bacterial colonisation, (4) selective cytotoxicity to tumour cells. SCFA short-chain fatty acids, CXCR4 CXC cytokine receptor 4, MHC-1 major histocompatibility complex class I, Th17 T helper cell 17, T-reg, T-regulatory cell, NF-κB, nuclear factor-kappa light-chain enhancer of activated B cells, STAT3 signal transducer and activator of transcription 3. Created with BioRender.com.

The GM has been linked to the development of a number of predominantly gastrointestinal and hepatobiliary malignancies including oesophageal [24], liver [25], pancreatic [26] and, most notably, colorectal cancer (CRC). CRC is the third most prevalent cancer worldwide and is associated with significant morbidity and mortality, having been the second leading cause of cancer death globally in 2018 [27]. Incidence rates have been rising in individuals <50 years old and this has been linked to alcohol, smoking, obesity, diabetes and physical inactivity [27]. Of particular interest, the European Prospective Investigation into Cancer and Nutrition (EPIC) study in 2016 validated findings that a diet rich in processed foods, animal fats and red meat, coupled with a low intake of fibre and fruits, was an important risk factor for developing sporadic CRC [28]. Pathogenesis involves a multistep process with accumulating genetic alterations in association with morphological changes and genetic instability. Of note, distinct metagenomic and metabolomic shifts have been identified in various stages of CRC pathogenesis, including in polypoid adenomas, intramucosal carcinomas and more advanced, metastatic lesions. They found a relative abundance of *Fusobacterium nucleatum*, which increased continuously from intramucosal carcinomas to more advanced stages (*p* < 0.005). Furthermore, *Atopobium parvulum* and *Acintomyces ondontolyticus* were significantly increased only in multiple polypoid adenomas and intramucosal carcinomas, compared to solitary lesions (*p* < 0.005). This large cohort study (*n* = 616) suggests that microbial and metabolomic changes may occur very early in the pathogenesis of CRC, which may be of clinical and diagnostic relevance [28]. Figure 2 illustrates the complex pathways putatively identified in maintaining GM homeostasis by beneficial commensal bacteria and probiotics, and the potential relationships between colorectal carcinogenesis and anti-tumour effects [29].

The GM supports a variety of mechanisms aimed to support probiotic growth and resist enteropathogenic colonisation independent of immune function, including the formation of bactericidal or bacteriostatic small molecules (bacteriocin from *Enterococcus faecalis* microcin by *Escherichia coli* Nissle), antimicrobial peptide induction (lugdunin from *Staphylococcus lugdunensis*) and, crucially, fermentation of dietary fibres into short-chain fatty acids (SCFAs). Colonocytes use the three major SCFAs, namely, acetate, propionate and butyrate, as energy sources, while transformed CRC cells primarily undergo aerobic glycolysis. Compared to normal colonocytes, CRC cells exhibit an increased sensitivity to SCFAs, demonstrating that they likely have a vital role in cell homeostasis [30]. Manipulation of SCFA levels in the gut, through changes in the GM, has therefore emerged as a potential preventive/therapeutic strategy for CRC. Several SCFA transporters like sodium-coupled monocarboxylate transporter-1, monocarboxylate transporter-1 (MCT-1) and aquaporins have been identified as the main transmembrane transporters in intestinal cells [31]. It has recently been shown that MCT-1 promotes tumour metastasis independent of its activity as a lactate transporter [32].

Gut mucosal dysfunction has also been implicated in colorectal carcinogenesis by the induction of epithelial-to-mesenchymal (EMT) transition and subsequent increase in gut permeability through the loss of tight-junction proteins such as zona occludins (ZO) 1 and 2, claudin-1 and occludin [33]. This may result in the translocation of bacteria and pathogenic metabolites, increasing the risk of local and systemic inflammation and subsequent carcinogenesis. In recent years, several species of bacteria have been identified to play a causal role in CRC pathogenesis by facilitating a detrimental intestinal microbial ecosystem. These species include, but are not necessarily limited to, *F. nucleatum*, *Streptococcus gallolyticus* (formerly known as *Streptococcus bovis* type 1), enterotoxic strains of *Bacterioides fragilis* (produces *B. fragilis* toxin BFT), polyketide synthase-positive (PKS^+ve^) strains of *E. coli* (produces colibactin), *E. faecalis* and *Peptostreptococcus anaerobius*, which have been implicated in tumour proliferation [34], induction of pro-inflammatory states [35] and evasion of anti-tumour immunity [34]. In particular, bacteraemia and endocarditis associated with *S. gallolyticus* have been linked to an increased risk of CRC in observational studies. Colibactin, a genotoxic polyketide-peptide produced in the gut by PKS^+ve^ *E. coli*, has been shown in murine models to induce DNA strand breaks, promote tumour formation, induce chromosomal aberrations, alter cell cycle behaviour, and increase mutation frequency rates [36, 37]. *Bacterioides fragilis*, on the other hand, has been linked to early neoplastic changes through the production of the metalloproteinase enterotoxin BTF, which is thought to induce cleavage of E-cadherin, a cell adhesion molecule, leading to an increase in paracellular permeability and activation of β-catenin resulting in increased cellular proliferation [38, 39]. Moreover, some bacteria, such as *Lachnospiraceae* species, *Bifidobacterium animalis* and *Streptococcus thermophilus*, are found to be depleted in CRC patients [40, 41].

*Fusobacterium nucleatum*, a well-known peridontal commensal and opportunistic pathogen, became relevant in the context of cancer as a result of sequencing-based studies of human CRC samples [42, 43], and mechanistic data from pre-clinical models that demonstrated a multitude of roles. In vitro, *F. nucleatum* promotes CRC cell proliferation, and in mouse models, promotes tumour growth in patient-derived CRC xenografts, modulated by the *F. nucleatum* adhesin, FadA, which binds to E-cadherin on the CRC cell surface and activates oncogenic Wnt/β-catenin signalling, thereby promoting cell proliferation [44, 45]. *Fusobacterium nucleatum* can also inhibit immunosurveillance by suppressing the cytotoxic functions of tumour-infiltrating lymphocytes and natural killer (NK) cells by binding to the inhibitory immune receptor TIGIT through another adhesin, Fap2 [46]. *Fusobacterium nucleatum* may also play a role to facilitate resistance to chemotherapy, with a large cohort study associating an abundance of *F. nucleatum* with reduced overall survival (OS) [47]. Functional investigations later revealed the role of *F. nucleatum* in promoting chemoresistance in CRC patients by activating autophagy through Toll-like receptor 4 (TLR4) expressed on CRC cells, rendering those tumours more resistant to oxaliplatin-induced cell death, with consequent treatment failure [46].

An in-depth discussion of the GM and its complex relationship with CRC pathogenesis and potential modulation is beyond the scope of this article, but is elegantly reviewed by Fong et al. [29], while a separate article by Brennan and Garrett undertakes a critical reappraisal of Fusobacteria with a focus on *F. nucleatum* as a mutualist, infectious agent and oncobacterium [46]. Whilst mechanistic and supportive evidence from human and animal studies are encouraging, a recent consensus statement published by the International Cancer Microbiome Consortium in 2019 concluded that there is currently no direct evidence that the human commensal microbiome is a key determinate in the aetiopathogenesis of cancer, and data from high-quality longitudinal cohort studies are needed to confirm this role [48].

### The GM and immune checkpoint inhibition

Therapeutic targeting of immune checkpoints, such as programmed death-ligand 1 (PD-L1), programmed cell death protein-1 (PD-1) and cytotoxic T-lymphocyte-associated protein-4 (CTLA4) with an immune checkpoint inhibitor (ICI), has revolutionised the treatment landscape of multiple malignancies, with several landmark randomised control trials showing significant survival benefits, resulting in changes to standard of care in specific patient subtypes [49, 50]. However, the efficacy of ICIs varies considerably between different cancer types. Several host genetic and immune factors, in addition to tumour-related biomarkers, have been elucidated to dictate response [51–54]. *Nature* deemed the influence of the GM on ICI in cancer to be amongst the five greatest discoveries of 2018 [55] following a number of seminal publications that we will discuss.

In 2015, two studies suggested the potential involvement of the GM in modulating the efficacy of anti-CTLA4- and anti-PD-1-based therapies [56, 57]. Vétizou et al. demonstrated that the efficacy of anti-CTLA4 antibodies in reducing sarcoma tumour growth in mice is significantly increased when the GM is enriched with *B. fragilis* and *Burkholderia cepacia* [56]. Furthermore, Sivan et al. found the efficacy of anti-PD-L1 antibodies in melanoma is improved in the presence of a GM enriched with *Bifidobacterium* spp. in murine models [57]. In addition, they demonstrated that the oral administration of *Bifidobacterium* spp., combined with anti-PD-L1 antibodies, specifically boosts T cell responses with augmented dendritic cell function, leading to enhanced CD8^+^ T cell priming and accumulation in the tumour microenvironment, inhibiting melanoma growth [57].

Multiple translational studies were published in 2018, which further supported the role of the GM in modulating the response to ICIs [58–60]. Routy et al. found that melanoma patients treated with antibiotics, alongside anti-PD-1/anti-PD-L1 ICI, had a lower survival rate, and metagenomic analyses of patients’ faecal GM showed a compositional difference [58]. Anti-PD-1 responders were enriched in two phyla, *Akkermansia* and *Alistipes*. Performing faecal microbiota transplantation (FMT) from patients to germ-free mice, the authors found that *Akkermansia muciniphila* increased intra-tumoral cytotoxic T cell infiltrates, thus increasing PD-1 blockade response in mice [58]. In parallel, Gopalakrishnan et al. demonstrated, through shotgun metagenomic sequencing of faecal samples from melanoma patients, that the anti-PD-1 responders’ GM was different in composition compared to non-responders [59]. The authors observed an increase in the abundance of *Clostridiales* and *Ruminococcaceae* amongst responders. Functional studies performed with FMT in germ-free mice further demonstrated how inoculating mice with the identified bacteria, along with the anti-PD-1 therapy, enhanced the anti-cancer effects and inhibited melanoma growth [59]. Matson et al. performed metagenomic characterisation of stool samples from melanoma patients treated with ICI, further corroborating the finding that the stools of responders have a different GM compared to those of non-responders [60]. They identified and proved in vivo the functional importance of *Bifidobacterium longum*, *Enterococcus faecium* and *Collinsella aerofaciens* in ameliorating anti-PD-L1 efficacy [60]. Gharaibeh and Jobin re-analysed the sequencing data of the three aforementioned studies [58–60] with the same pipeline and demonstrated that the analytical pipeline did not drive heterogeneity in microbial signals across these studies [61]. In their analysis, microbial gene content had a greater predictive power and shared signal compared to microbial composition, suggesting that microbial signals are intrinsic to each study but may be functionally related. Additional groups have performed similar studies, identifying further species associated with a potential response to ICI [62]; a number of these single small datasets were recently meta-analysed [63] to verify the partially conflicting biomarkers of response to ICI across different patient cohorts [59, 60, 62, 64–66]. They identified both known microbial features enriched in ICI responders, including the prevailing taxa *Faecalibacterium*, and additional features including an over-representation of *Barnesiella intestinihominis* and vitamin B metabolites. Crucially, they were able to predict ICI responders in an independent cohort (*n* = 27), which was also predictive of prognosis. It is clear that the role of the GM appears far more complex than previously thought, extending beyond specific species and functions present in responders and non-responders, and suggests the existence of a unique, multi-mechanistic interplay of biological factors, which may be reflected in the faecal microbiome signatures, offering the potential for therapeutic and diagnostic exploitation.

Treatment-related toxicity remains a major challenge with ICI, frequently leading to delay and discontinuation of treatment [67]. Patients receiving combination anti-CTLA4 and anti-PD-1 blockade are counselled that their risk of high-grade toxicity requiring hospital admission exceeds 50%, based on relevant studies [50]. In pre-clinical models, oral gavage of *B. fragilis* and *B. cepacia* demonstrated an amelioration of such immune-related adverse events (irAEs) [56]. In line with this observation, it has been shown in patients treated with anti-CTLA4 antibodies that irAEs are mediated by an increased abundance of *Firmicutes* and a decreased abundance of *Bacterioides* [62, 68]. Altogether, this suggests a strong association between the composition of the GM in modulating the effects of both ICI response and irAEs.

However, many important questions remain—the most obvious of these relating to mechanisms. ICI responses likely occur at least in part because their microbiota gives patients a pre-existing immune response that is amplified by ICI, and the microbiota may potentially prime cells for an effective immune response. For example, bacterial species *B. animalis*, *Lachnospiraceae* spp. and *S. thermophilus* were found to be depleted in CRC patients, and are thought to exert a protective effect against CRC carcinogenesis [29, 41, 69]. However, it is uncertain whether a favourable response can be tied to a single bacterium or even a specific combination of species. Recently, Mager et al. discovered that *Bifidobacterium pseudolongum* produced a metabolite called inosine, which enhanced the effect of ICI in mouse models [70]. The effect of inosine was dependent on T cell expression of the adenosine A_2A_ receptor and required co-stimulation. Moreover, Wang et al. have recently demonstrated in elegant pre-clinical studies that inosine supplementation enhances the anti-tumour efficacy of ICI and adoptive T cell transfer in solid tumours defective in metabolising inosine, demonstrating the capability of inosine to relieve tumour-imposed metabolic restrictions on T cells [71]. Metabolites such as inosine represent the functional output of the GM, and conceivably may be shown to be more exploitable than bacterial taxa in the development of microbial-based therapies.

### The GM and stem cell transplantation

There is mounting evidence for the considerable effect of the human intestinal microbiome on the clinical course following haematopoietic stem cell transplantation (HSCT) [72–74]. Microbial abundances in the intestinal ecosystem may be potential biomarkers or therapeutic targets for preventing relapse and improving survival rates after HSCT [75]. Recent work has demonstrated the potential of the microbiota to be used as a predictor of mortality in allogeneic HSCT [75]. In addition, studies have shown that targeted modulation of the GM in HSCT patients may have potential therapeutic implications [76]. Complications such as graft-versus-host disease (GVHD) remain a major cause of illness and death, limiting the broader applicability of allogeneic haematopoietic cell transplantation; a number of pre-clinical studies have shown that commensal bacteria influence the pathophysiology of GVHD [10, 17–20]. Two different strategies with targeted modulation of the GM, pre-emptive and therapeutic, have been used for the prevention and treatment of imbalances of microbial ecosystems in patients with HSCT, succinctly summarised by Yu et al. [77] and many of these are currently being investigated in clinical trials.

### The GM and other anti-cancer therapies

In the majority of patients with advanced malignancies, cytotoxic chemotherapy remains the mainstay of treatment. Investigations into the relationship between the GM and chemotherapy remain scarce, and the majority of available data is pre-clinical. However, there is some emerging evidence to suggest a link  between the GM and chemotherapy efficacy, toxicity and failure. In either germ-free tumour-bearing mice or mice that have had their GM depleted with antibiotics, responses to oxaliplatin were found to be inefficient compared to those in which the GM was left intact [78]. Mechanistically, it has been hypothesised that commensal microbiota act by regulating TLR agonists, which promote a rise in reactive oxygen species (ROS) with subsequent tumour cell death [78]. Consistently, lung-tumour-bearing mice treated with cisplatin and GM-depleting antibiotics were shown to develop larger tumours and have poorer survival. Yet, a combination of cisplatin with probiotics such as Lactobacillus showed an improved response to therapy. The induction of pro-apoptotic genes and the enhancement of immunosurveillance may play a key role in this finding [79]. When combined with oral administration of *Lactobacillus johnsonii* and *Enterococcus hirae*, cyclophosphamide therapy led to the conversion of naive T cells to pro-inflammatory Th17, with the final effect of improving cyclophosphamide efficacy in tumour-bearing mice [80].

Furthermore, the GM has been implicated in chemotherapy-induced neurological toxicities, including the development of peripheral neuropathy, cognitive impairment and psychological sequelae [81, 82]. The development of chemotherapy-induced neuropathic pain (CINP) is thought to be modulated by systemic translocation of bacterial metabolites across a compromised mucosal barrier to modulate the gut–immune axis, resulting in altered central pain processing, and the potentiation of CINP. Paclitaxel, in this instance, has been implicated by decreasing the abundance of favourable *A. muciniphila*, resulting in the impairment of mucosal barrier integrity and allowing for pathognomonic translocation of pro-inflammatory and neuromodulatory metabolites [83].

## How do medications affect the GM?

### Antibiotics

A 2019 review on the use of antibiotics in immuno-oncology described how 11 of 12 included studies demonstrated a negative impact of antibiotics on clinical outcomes in patients receiving ICIs for non-small cell lung cancer (NSCLC), renal cell carcinoma (RCC) and melanoma [84]. Since then, a number of studies have been performed with the aim of answering deeper questions of mechanism. One such study by Derosa et al. prospectively collected stool samples from 69 advanced RCC patients treated with the ICI nivolumab. Recent antibiotic use reduced objective response rates (from 28 to 9%, *p* < 0.03) and markedly affected the composition of the microbiota via shotgun metagenomic sequencing, facilitating the dominance of distinct species such as *Clostridium hathewayi*, which were also over-represented in stools from RCC patients compared with healthy volunteers [85]. Hakozaki et al. prospectively collected pre-ICI stool samples from 70 Japanese patients with advanced NSCLC and treated them with anti-PD-1/PD-L1 ICI [86]. Using 16S RNA gene sequencing, bacterial diversity and differential abundance analysis was performed. Patients who received pre-ICI antibiotics had lower α-diversity at baseline and under-representation of *Ruminococcaceae* UCG 13 and *Agathobacter*. When analysing antibiotic-free patients, α-diversity correlated with OS. In addition, *Ruminococcaceae* UCG 13 and *Agathobacter* were enriched in patients with favourable objective response rates and progression-free survival (PFS) >6 months. While we know that GM reconstitution can take many months, it remains unknown whether there is an ideal time-point prior to treatment in which antibiotic exposure adversely affects outcomes. In a pre-clinical study, a 10-fold decrease in the bacterial load and reduced bacterial phylotype diversity was seen after 3 days of broad-spectrum antibiotics [87]. In humans, Corbeil and colleagues demonstrated exposure to a second-generation cephalosporin over a period of 7 days led to a loss of metagenome-defined species [88]. Another study of 370 patients examined the impact of antibiotics within 4–8 weeks before ICI was commenced and reported that the impact of antibiotics on the clinical efficacy of ICI was less marked when antibiotics were not delivered within 30 days of initiation [89]. Furthermore, a recently published study of 196 patients receiving ICI demonstrated that patients who received antibiotics within 1 month of treatment initiation had significantly worse OS compared to those who received antibiotics whilst concurrently receiving ICI [90]. Indeed, prospective studies to validate antibiotic-mediated gut perturbations as a mechanism of ICI refractoriness, and to guide the development of strategies to overcome this barrier to optimise ICI responses, are clearly required.

### Other medications

Many non-antibiotic medications can also have harmful effects on commensal gut microbiota. Proton pump inhibitors (PPIs) have been the most well studied and their long-term use has been linked to an increased gastric cancer risk [91]. A number of groups have demonstrated increased *Streptococcaceae* and *Micrococcaceae* abundance in PPI users [92, 93]. While it has been shown that long-term PPI use is associated with an increased risk of gastric cancer even after *Heliobacter pylori* eradication [94], high-quality studies linking cancers, PPIs and specific microbiota are lacking. In the context of ICI, a retrospective multivariate analysis of 140 patients with advanced melanoma demonstrated that baseline PPI use almost halved objective response rates and reduced PFS and OS of patients treated with combination ipilimumab and nivolumab [95].

Corticosteroid use in the context of ICI is of particular relevance, as corticosteroids are frequently used to manage irAEs. However, corticosteroid use has been shown to be an independent risk factor for survival in the setting of ICI. Among a cohort of 640 NSCLC patients treated with ICI, those who had continuously received corticosteroids of ≥10 mg of prednisone-equivalent daily had a reduced PFS and OS, as confirmed by both univariate and multivariate analysis [96]. Beyond anti-inflammatory and immunosuppressive effects, corticosteroids may cause substantial shifts in the GM ecology. For instance, dexamethasone causes an increase in the abundance of *Clostridiales* and *Lactobacillaceae* in murine models; however, such data are lacking in humans at present [97].

## Can we modulate the GM?

**Fig. 3:**
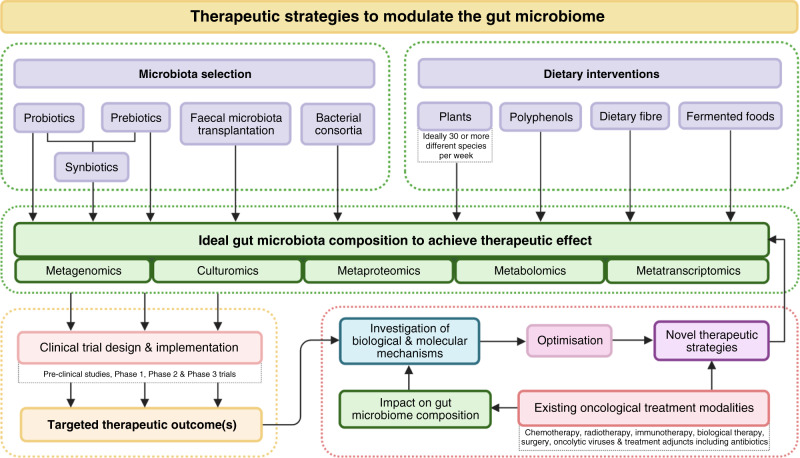
Summary of the current therapeutic strategies to modulate the gut microbiome in relation to translational research. Created with BioRender.com.

The therapeutic potential to improve patient outcomes by manipulating the GM is an exciting one. However, despite decades of research, the composition of an “ideal” GM remains unknown. While favourable and unfavourable bacteria have been identified, the complexity of the GM, coupled with our limited understanding of the vast number of complex interactions, may prove that the simple identification of microbes represents only the tip of the iceberg. There is unlikely to be a one-size-fits-all GM, and the ideal GM of any particular individual may be dependent on a number of modifiable and non-modifiable factors (Fig. 1). Globally, many interventional studies are currently underway focusing on ICI in various cancers as well as other human diseases. Crucial to this is an integrated understanding of how existing oncological treatments impact microbiome-based interventions. This is illustrated in Fig. 3, which summarises the complex relationship between GM-based treatment interventions and the ongoing need for scientific innovation through proof-of-concept studies, molecular and biological research and omics analyses—with the aim of accelerating and optimising novel and existing therapeutic strategies to shape the ideal, therapeutic GM, which in turn can be validated in high-quality clinical trials.

### Faecal microbiota transplantation

FMT is the process by which the liquidised stool (or its cryopreserved microbial contents) of a healthy donor is introduced into the colon of another individual through acid-resistant capsules, a nasogastric tube or rectally. Theoretically, faecal samples could be prepared from either anti-PD-1 responders showing a favourable GM or simply a relatively young and healthy person with a diverse GM, and then transplanted into anti-PD-1 resistant patients [98]. In *Clostridioides difficile* infection (CDI), FMT has been used successfully to manage treatment-resistant CDI colitis [99]. Moreover, FMT has been used in the management of GVHD following allogeneic stem cell transplantation [100]. Pre-clinical murine studies have demonstrated the efficacy of FMT in reducing colorectal tumorigenesis; however, further research is required to determine whether this can be replicated in humans [101]. Recently, a Phase 1 clinical trial assessed the safety and feasibility of FMT and reinduction of anti-PD-1 ICI in 10 patients with anti-PD-1-refractory melanoma [102]. Three patients responded (two partial responses and one complete response). Of note, FMT was associated with favourable changes in immune cell infiltrates and gene expression profiles in both the gut lamina propria and the tumour microenvironment. In addition, a small number of case reports of FMT successfully treating ICI-associated colitis have been published [103, 104], representing an area of potential research interest.

### Probiotics, synbiotics and bacterial consortia

It is possible that beneficial immune-potentiating bacteria could be prepared as therapeutic adjuncts or even independent anti-cancer agents for administration to patients. *Probiotics* are live bacteria that are thought to provide health benefits and aid in GM homeostasis, while *synbiotics* are a combination of prebiotics and probiotics in a single formulation. *Bacterial consortia* are formed from the combination of two or more microbial groups to create a defined mixture with the aim of harnessing specific functions of individual microbiota, or synergistic effects of multiple microbiotas, to be administered as an intervention with therapeutic potential. Historically, some difficulties have been shown to exist in the transportation of live bacteria beyond the acidic environment of the upper GI tract, but efforts are underway to combat this. Table 1 shows a number of clinical trials underway to treat patients with various solid tumours using therapeutic microbiome material (sometimes referred to as “next-generation probiotics” (NGPs), a concept that overlaps with the emerging concept of live biotherapeutic products (LBPs)) in combination with conventional ICI. These are all early phase trials, designed to generate early data on product formulations, doses and safety. It is worth noting that while NGPs may conform to the normal definition of a probiotic, they are more likely to be delivered within the US Food and Drug Administration (FDA) definition of an LBP. As such, the likely route to market for these LBPs/NGPs follows a path marked by studies of the pre-clinical mode of action, safety pharmacokinetics and pharmacodynamics and Phase 1–3 trials, accompanied by the standard regulatory approval hurdles. The increasingly complicated domain of LBPs, the regulatory frameworks they face and how scientists must manage this are discussed by O’Toole et al. [105].

**Table 1:** Ongoing clinical trials examining microbial therapeutics alone or in combination with ICI for cancer.

Clinical trial ID	*N* = ; study type	Start; estimated completion date	Condition or disease	Intervention
NCT03817125*	10; Phase Ib multicenter SER-401/placebo 2:1 randomised, placebo-controlled, blinded study	January 2019; January 2023 *Discontinued March 2021 due to enrolment impact by COVID19	Advanced melanoma (unresectable or metastatic)	SER-401 (donor-derived, enriched in *Rumminococcacacae*) combination treatment with anti-PD-1 ICI nivolumab compared to matching placebo
NCT03637803	132; Phase I/II Open-label, safety and preliminary efficacy study	January 2019; March 2024	Anti-PD-1/PD-L1 relapsed advanced solid tumours (NSCLC, RCC, bladder cancer and melanoma)	MRx0518 (*Enterococcus gallinarum*) combination treatment anti-PD-1 ICI pembrolizumab
NCT03934827	120; Phase I Safety study; Part A Open-label preliminary safety study; Part B MFx0518/placebo 4:1 randomised, double-blinded study	April 2019; February 2022	Solid tumours awaiting primary surgical resection	MRx0518 (*E. gallinarum*) compared to placebo given until surgical resection (Experimental Arm Part B)
NCT03775850	120; Phase I/II Open-label 3-Cohort study	December 2019; December 2020	Advanced metastatic CRC, triple-negative breast cancer and ICI relapsed solid tumours	EDP1503 (*Bifidobacterium animalis lactus*) given alone for 14 days, followed by combination treatment with anti-PD-1 ICI pembrolizumab
NCT03595683	70; Phase II Dual-Cohort, Open-label study	October 2018; November 2023	PD-1 relapsed and naive advanced melanoma	EDP1503 (*B. animalis lactus*) combination treatment with anti-PD-1 ICI pembrolizumab
NCT03686202	65; Early Phase I Open-label, randomised, feasibility study	September 2018; April 2021	All solid tumours (locally advanced and metastatic)	MET-4 (defined consortium from donor-derived intestinal bacteria) combination treatment with SOC ICIs
NCT04208958	111; Phase I/II Open-label single-group assignment study	January 2020; April 2022	Advanced metastatic cancer; melanoma, gastric/gastroesophageal junction (GEJ) adenocarcinoma, microsatellite-stable (MSS) CRC	VE800 (11 strain defined consortium) combination treatment with anti-PD-1 ICI Nivolumab

Sourced from Clinicaltrials.gov from the National Library for Medicine (NLM) at the National Institutes of Health (NIH), US Department of Health and Human Services.

Many patients currently take commercially -available, “conventional” probiotics, which are generally drawn from a narrow range of genera, which mainly include *Lactobacillus* spp. and *Bifidobacterium* spp. However, the clinical evidence surrounding these conventional probiotics does not appear to confer any significant health benefits, especially amongst healthy individuals [106]. In fact, recent studies have expressed concerns that probiotic use after antibiotic administration could delay the restoration of the GM [107]. In the context of cancer, a number of human studies have suggested non-specific probiotic intake for patients receiving ICI may actually be detrimental; one small study of 46 melanoma patients presented at the American Association for Cancer Research annual conference in 2019 showed that the consumption of conventional probiotic supplements (purchased from pharmacies or online) was associated with a 70% reduced response rate to ICI [108], although this evidence has not yet been published and, therefore, should be interpreted with caution. Another study of 312 melanoma patients receiving ICI reported 42% of patients were taking probiotics and this group was found to have lower GM diversity, which was associated with inferior responses and reduced survival [109]. In addition, although such agents are generally regarded as safe, there are emerging concerns around the administration of live bacteria to immunocompromised individuals, with the potential risk of opportunistic infections, as well as the transfer of antibiotic resistance [110]. Despite this, probiotic administration in multiple trials has shown beneficial effects in ameliorating chemotherapy-induced diarrhoea and other gut-related side effects, which in part is thought to be due to the establishment of a healthy and resilient GM [111]. Furthermore, major international bodies in oncology including the MASCC/ISOO (Multinational Association of Supportive Care in Cancer and International Society of Oral Oncology) and ESMO (European Society of Medical Oncology) now formally recommend probiotic administration with *Lactobacillus* spp. to prevent diarrhoea in patients receiving chemotherapy and/or radiation therapy for pelvic malignancies [112].

Synbiotics and bacterial consortia represent emerging areas of probiotic and microbiome-based treatments. In a pre-clinical rodent model, a synbiotic cocktail upregulated gene associated with tight-junction formation and mucous production and inhibited the development of CRC [113]. Furthermore, a synbiotic preparation containing *Lactobacillus casei* has been shown to enhance NK cell activity in rodent models [114]. A randomised control trial, which compared synbiotic administration to placebo in polypectomised or CRC patients, demonstrated an improvement in several CRC biomarkers, such as DNA damage and cellular proliferation, in the synbiotic group including interleukin-2 and interferon-γ (*t*_3_) [115].

Due to synergy and interactions such as horizontal gene transfer, cross-feeding and inter-signalling, bacterial consortia may provide a physiological advantage when compared to single organisms [116]. For example, VE800 is a collection of 11 commensal gut bacterial strains shown in pre-clinical models to be capable of robustly inducing interferon-γ-producing CD8^+^ T cells in the intestine [117]. An open-label clinical study (NCT04208958) is currently exploring the safety, tolerability and clinical activity of VE800 in combination with nivolumab in patients with advanced malignancies (Table 1).

### Dietary change

Dietary interventions have the advantage of being safe, cost-efficient, and readily accessible. A change in diet can alter the composition of the GM, with evidence showing significant microbial shifts within 5 days of commencing dietary interventions [118]. While easily modified, complexities around compliance, administration schedules and uncertainty around sustained benefits in any GM changes exist. Everything that we ingest has the potential to alter the composition of our microbiome, but we will focus on dietary fibres, prebiotics and probiotics, polyphenols, fermented foods, animal meat and sweeteners and preservatives.

#### Dietary fibres and prebiotics

Dietary fibres are edible carbohydrate polymers with ≥3 monomeric units resistant to endogenous digestive enzymes. Prebiotics are food components (mostly dietary fibres) or ingredients that are not digestible by the human body, but specifically or selectively nourish beneficial colonic microbes. Dietary fibre is not hydrolysed by human digestive enzymes, but acted upon by gut microbes resulting in metabolites such as SCFAs. Average dietary fibre consumption amongst healthy individuals in the United Kingdom is less than half of the recommended 30 g/day [119]. Such low fibre intakes reduce the production of SCFAs and shifts the GM metabolism to utilise less favourable nutrients [120], which may result in the production of potentially detrimental metabolites. The American Gut Project has demonstrated that people who consume ≥30 different plant species (many of which have prebiotic properties) weekly have the healthiest and most diverse microbiomes [121], regardless of whether they also ate dairy, fish or red meat. An unpublished study examined the effect of diet and amongst 46 patients receiving anti-PD-1 ICI for melanoma and demonstrated that patients reporting high-fibre diets were approximately 5 times more likely to respond to therapy when compared to those with low-fibre diets [108]. However, as with all unpublished data, such results should be approached with caution and should not be overinterpreted, at least until published—at which point methods and potential biases can be closely examined and critiqued.

#### Polyphenols

Polyphenols are compounds containing more than one phenolic hydroxyl group and make up the biggest group of phytochemicals. They are found abundantly in a wide variety of foods, such as colourful fruits, vegetables, herbs, seeds, cereals, coffee, tea, cocoa and wine [122]. Phenolic compounds and their metabolites are thought to contribute to beneficial gastrointestinal health effects by modulating gut microbial balance with the simultaneous inhibition of pathogens. Consumption of cocoa-derived polyphenols has been linked with significant increases in plasma high-density lipoprotein and significant reductions in plasma triglyceride and C-reactive protein concentrations [123]. Reductions in pathogenic *Clostridium* spp. have been noted after consumption of fruit, seed, wine and tea polyphenols [123].

#### Fermented foods

Fermented products (e.g., kefir, many yoghurt products, sauerkraut, sourdough breads, kimchi, kombucha) are made by microbial organisms and enzymatic conversions of major and minor food components and have garnered some optimism due to their potential to prevent and manage a range of disease, including cancer [124]. A meta-analysis of 61 studies with >1.9 million participants demonstrated statistical evidence supporting an association with fermented dairy food intake and an overall decrease in cancer risk [124]. Associations varied, with yoghurt consumption associated with decreased bladder cancer and CRC risk, but an increase in prostate and renal cancer risk. Other meta-analyses have shown protective effects of yoghurt consumption on breast cancer [125].

#### Sweeteners and preservatives

High-intensity sweeteners are commonly used as low-calorie sugar alternatives. Despite being “generally recognised as safe” by regulatory agencies including the FDA, animal studies have shown that these substitutes may have negative effects on the gut microbiota [126]. Suez et al. demonstrated non-caloric artificial sweeteners (NAS) to alter microbial metabolic pathways linked to host susceptibility to metabolic disease and showed similar NAS-induced microbiome perturbations and glucose intolerance in healthy human subjects [107]. Sucralose, aspartame and saccharin have been shown to disrupt microbiome balance and diversity in both humans and animal models [127]. Food additives, such as emulsifiers, which are ubiquitous in processed foods, have also been shown to affect the gut microbiota in animal models [128]. Mice fed relatively low concentrations of two commonly used emulsifiers showed reduced microbial diversity compared with controls. *Bacteroidales* and *Verrucomicrobia* were decreased and inflammation-promoting *Proteobacteria* was enriched [128].

#### Animal meat

The current literature supports a 15–20% increased risk of CRC with each 100 g of red meat or 50 g of processed meat consumed daily [129]. While plant protein has been observed to increase levels of intestinal SCFA levels associated with a favourable GM, counts of unfavourable bile-tolerant anaerobes such as *Bacteroides*, *Alistipes* and *Bilophila* have been noted to increase with animal meat consumption [118]. One study found that subjects with a high animal meat diet have reduced *Roseburia* and *Agathobacter rectalis* (formerly known as *Eubacterium rectale*) in their gut microbiota and a decreased proportion of butyrate in their faeces [130]. Overall, the current opinion suggests that meat-eaters in Western populations should reduce their processed meat consumption [131].

## What does this mean for our current patients?

Oncologists should remain mindful of the GM of their patients, and the potential effects of antibiotics and other medications on cancer therapy, particularly those on ICI where the majority of treatment-related data exists. Dietary change is the easiest, most non-invasive means by which the GM can be modified. Yet, nutritional interventions have been under-utilised especially in cancer patients who do not present with malnutrition. In considering the available evidence, it may be useful to advise patients on healthy dietary interventions, which may translate to potential benefits and clinical outcomes. We have previously published these recommendations [132], and summarise them in Table 2.

**Table 2:** Dietary and general recommendations for patients and physicians prior to commencement of ICI [133].

Dietary
• Patients to diversify their diets as much as possible, by aiming to consume a greater variety of food types and colours
• Patients should aim to meet their recommended daily fibre intake of 30 g/day
• Patients should aim to consume at least 30 plant species per week (includes nuts, seeds, herbs, grains, fruit and vegetables)
• Consumption of artificial flavours, sweeteners and additives should be minimised, as well as ultra-processed foods with multiple additives
• Where possible, patients should aim to eliminate processed meats and replace protein sources with nuts, mushrooms and legumes
• Where animal meats are consumed, patients should reduce excess meat-eating and purchase the highest quality meats they can afford
• Patients should be advised that data on extreme diets is lacking and sudden and significant changes in eating patterns could potentially be dangerous
• Patients should be advised against consumption of store-bought commercial probiotic supplements and where possible, regularly consume fermented foods containing live microbes, where appropriate
**General**
• Patients with cancer should have access to nutritional support through a qualified dietician or nutritionist, and side effects of dietary changes should be monitored
• Patients and their general practitioners should be advised that broad-spectrum antibiotic usage in the 3 months prior to, but particularly during the month before ICI initiation, should be avoided unless absolutely necessary clinically
• If antibiotics are deemed necessary, microbiology consultation and efforts to narrow the spectrum of antimicrobial cover should be considered
• Pending more complete data, future consideration may be given to temporary delay of initiation of non-urgent ICI (e.g. very low volume metastatic disease) if a patient has had broad-spectrum antibiotics within 1 month of planned treatment initiation to allow for reconstitution of the GM
• PPI treatment should be stopped in patients with cancer where there is no obvious indication for it. Where patients do have a requirement for gastric protection, consideration should be given to a switch to a histamine H2-receptor antagonist, as they have not been shown to induce the same dysbiosis as with PPIs

Given the complexities intrinsic to microbiome research, mechanisms of effect and proof of direct causation remain elusive; significantly larger sample sizes, more consistent means of sample collection and processing, and more time are required. Therefore, it is important to remain cautious on this topic when advising patients, particularly when making decisions regarding their treatment. It is now clear, however, that the era of oncological care where patients’ diets were largely focussed solely on caloric intake rather than dietary composition has long since passed. Acknowledging the current limitations of available data, we feel that it is fair to state that the composition of the GM *likely* matters far more within oncology than has been demonstrated to date, and while hard and specific advice cannot be given with confidence given a lack of high-quality evidence, the general advice is certainly possible. It is very likely that there is no one-size-fits-all solution; what is required to achieve and maintain a balanced microbial ecosystem likely differs between patients, disease states and potentially between malignancies. However, the available evidence clearly shows that diversity of diet, particularly with respect to plants (fruits, vegetables, nuts, seeds and grains), consumption of “whole foods” and avoidance of both processed items and excessive red meat consumption, are all key components of a “GM friendly” diet—and we should advocate such a diet for those of our patients who can manage it. For those with advanced cancers, who are cachectic, or have difficulty tolerating an oral diet, the situation is more complex, and studies are needed in this space.

While we can be quite certain that antibiotics have a detrimental effect on the GM and may affect a particular patients’ chance at a response to ICI or other anti-cancer therapies, we must not overinterpret the data. We should always consider the clinical scenario in its entirety. Clearly, infections in unwell, neutropenic or frail cancer patients should be swiftly treated with appropriate antibiotics, and delays should not occur due to what are still somewhat theoretical risks of harm due to microbial imbalances. However, emerging and existing evidence should give pause for thought when the need for antimicrobials is in question. Prior to ICI initiation, in particular, antibiotics should be given only when there is a genuine indication—not, for example, for vague sore throats, minor skin ailments or other “soft” indications for which, in practice, they are often prescribed. These discussions are nuanced and require clinical acumen above all else; we greatly hope that the next few years of microbiome research in oncology provides the evidence to formulate specific and detailed guidelines and protocols.

## Future directions and challenges for microbiome research in oncology

The GM may provide opportunities to improve patient care at various stages of the cancer journey from screening to diagnosis and risk stratification before and during treatment. The incongruence of beneficial microbiome signatures across studies, along with an emerging understanding of the mechanisms underlying the interactions between the microbiome, metabolome and host immune system, highlight a critical need for additional comprehensive and standardised multi-omics studies. Numerous ICI studies have led to interventional trials aiming to modulate the GM and improve responses in ICI patient groups, but further research is required across other tumour subtypes, including those with fewer current links to the GM. It is clear that large prospective clinical trials incorporating nutritional assessments are needed.

Given the highly personalised responses seen between individuals with respect to diet [133], and the close association between diet and the GM, a personalised approach, based on specific patients’ GM composition, may be required. The holy grail of onco-microbial science has been determining predictive microbial biosignatures for various disease states with high levels of accuracy. Indeed, analysis of the GM in the context of human disease has predominantly been focused on the individual microbial composition rather than the functional output, particularly in the context of cancer. Identification of key metabolic markers and pathways may represent the functional output of the GM, and we predict that metabolomics will likely play a greater role in microbiome science over the coming years.

Altering the composition of the intestinal microbiota may predispose individuals to other health problems. In particular, there is mounting evidence of an interaction between the intestinal microbiota, the gut and the central nervous system and FMT is currently being trialled as a novel therapy targeting a range of psychiatric diagnoses including depression and anxiety, as reviewed by Meyyappan et al. [134]. Clearly, if FMT from a healthy individual to an individual with a psychiatric illness can provide therapeutic benefit, the converse may also be true, with a potential risk of inadvertently predisposing, or, at worst, cause, an FMT recipient to develop a psychiatric sequelae. In addition, many infections of immunocompromised/neutropenic patients originate from the gastrointestinal tract. The pathogenesis of these infections is often poorly understood, but likely initiates with their alteration or disruption through antibiotic use and the impairment of host immunity. It remains clear that more research is required into the role of GM modulation in the context of immunosuppression.

## Conclusion

Overall, the last decade has demonstrated the importance of the GM on cancer pathogenesis, progression, sustenance and treatment outcomes. However, microbiomes are complex ecosystems with spatiotemporal dynamics arising from interactions between microbes and host cells. Each cancer itself is an evolving ecosystem in which interactions occur with neighbouring cancer cells, stromal cells and the tumour microenvironment. As such, determining predictive microbial biosignatures, which may be of clinical benefit through GM sampling, metagenomic sequencing and bioinformatics, has a major role to play in the future of gut microbial research. Whilst major international studies are currently underway to elucidate the impact of GM on cancer therapy, it is clear that onco-microbial science is just beginning.

## Data Availability

Not relevant.

## References

[1] Bull MJ, Plummer NT (2014). Part 1: the human gut microbiome in health and disease. Integr Med (Encinitas).

[2] Nagpal R, Mainali R, Ahmadi S, Wang S, Singh R, Kavanagh K (2018). Gut microbiome and aging: physiological and mechanistic insights. Nutr Healthy Aging..

[3] Tordesillas L, Berin MC (2018). Mechanisms of oral tolerance. Clin Rev Allergy Immunol.

[4] Johansson ME, Jakobsson HE, Holmén-Larsson J, Schütte A, Ermund A, Rodríguez-Piñeiro AM (2015). Normalization of host intestinal mucus layers requires long-term microbial colonization. Cell Host Microbe.

[5] Huda MN, Lewis Z, Kalanetra KM, Rashid M, Ahmad SM, Raqib R (2014). Stool microbiota and vaccine responses of infants. Pediatrics..

[6] Tang WHW, Hazen SL (2014). The contributory role of gut microbiota in cardiovascular disease. J Clin Invest.

[7] Wang Y, Kasper LH (2014). The role of microbiome in central nervous system disorders. Brain Behav Immun.

[8] Yacoub R, Jacob A, Wlaschin J, McGregor M, Quigg RJ, Alexander JJ (2018). Lupus: the microbiome angle. Immunobiology..

[9] Zhao L (2013). The gut microbiota and obesity: from correlation to causality. Nat Rev Microbiol.

[10] Frosali S, Pagliari D, Gambassi G, Landolfi R, Pandolfi F, Cianci R (2015). How the intricate interaction among Toll-like receptors, microbiota, and intestinal immunity can influence gastrointestinal pathology. J Immunol Res.

[11] Levy M, Kolodziejczyk AA, Thaiss CA, Elinav E (2017). Dysbiosis and the immune system. Nat Rev Immunol.

[12] Carroll IM, Chang YH, Park J, Sartor RB, Ringel Y (2010). Luminal and mucosal-associated intestinal microbiota in patients with diarrhea-predominant irritable bowel syndrome. Gut Pathog.

[13] Rangel I, Sundin J, Fuentes S, Repsilber D, de Vos WM, Brummer RJ (2015). The relationship between faecal-associated and mucosal-associated microbiota in irritable bowel syndrome patients and healthy subjects. Aliment Pharmacol Ther.

[14] Swidsinski A, Loening-Baucke V, Verstraelen H, Osowska S, Doerffel Y (2008). Biostructure of fecal microbiota in healthy subjects and patients with chronic idiopathic diarrhea. Gastroenterology..

[15] Fouhy F, Deane J, Rea MC, O’sullivan Ó, Ross RP, O’callaghan G (2015). The effects of freezing on faecal microbiota as determined using MiSeq sequencing and culture-based investigations. PLoS ONE.

[16] Choo JM, Leong LE, Rogers GB (2015). Sample storage conditions significantly influence faecal microbiome profiles. Sci Rep.

[17] Menke S, Gillingham MAF, Wilhelm K, Sommer S (2017). Home-made cost effective preservation buffer is a better alternative to commercial preservation methods for microbiome research. Frontiers in Microbiology.

[18] Flores R, Shi J, Yu G, Ma B, Ravel J, Goedert JJ (2015). Collection media and delayed freezing effects on microbial composition of human stool. Microbiome..

[19] Tang Q, Jin G, Wang G, Liu T, Liu X, Wang B (2020). Current sampling methods for gut microbiota: a call for more precise devices. Front Cell Infect Microbiol.

[20] Brumfield KD, Huq A, Colwell RR, Olds JL, Leddy MB (2020). Microbial resolution of whole genome shotgun and 16S amplicon metagenomic sequencing using publicly available NEON data. PLoS ONE.

[21] Zierer J, Jackson MA, Kastenmüller G, Mangino M, Long T, Telenti A (2018). The fecal metabolome as a functional readout of the gut microbiome. Nat Genet.

[22] Singhal N, Kumar M, Kanaujia PK, Virdi JS (2015). MALDI-TOF mass spectrometry: an emerging technology for microbial identification and diagnosis. Front Microbiol.

[23] Lagier JC, Khelaifia S, Alou MT, Ndongo S, Dione N, Hugon P (2016). Culture of previously uncultured members of the human gut microbiota by culturomics. Nat Microbiol.

[24] Baba Y, Iwatsuki M, Yoshida N, Watanabe M, Baba H (2017). Review of the gut microbiome and esophageal cancer: pathogenesis and potential clinical implications. Ann Gastroenterol Surg.

[25] Yu L-X, Schwabe RF (2017). The gut microbiome and liver cancer: mechanisms and clinical translation. Nat Rev Gastroenterol Hepatol.

[26] Wei M-Y, Shi S, Liang C, Meng Q-C, Hua J, Zhang Y-Y (2019). The microbiota and microbiome in pancreatic cancer: more influential than expected. Mol. Cancer.

[27] Bray F, Ferlay J, Soerjomataram I, Siegel RL, Torre LA, Jemal A (2018). Global cancer statistics 2018: GLOBOCAN estimates of incidence and mortality worldwide for 36 cancers in 185 countries. CA Cancer J Clin.

[28] Gonzalez CA, Riboli E (2010). Diet and cancer prevention: Contributions from the European Prospective Investigation into Cancer and Nutrition (EPIC) study. Eur J Cancer.

[29] Fong W, Li Q, Yu J (2020). Gut microbiota modulation: a novel strategy for prevention and treatment of colorectal cancer. Oncogene..

[30] Gomes SD, Oliveira CS, Azevedo-Silva J, Casanova MR, Barreto J, Pereira H (2020). The role of diet related short-chain fatty acids in colorectal cancer metabolism and survival: prevention and therapeutic implications. Curr Med Chem.

[31] Sivaprakasam S, Bhutia YD, Yang S, Ganapathy V (2017). Short-chain fatty acid transporters: role in colonic homeostasis. Compr Physiol.

[32] Payen VL, Hsu MY, Rädecke KS, Wyart E, Vazeille T, Bouzin C (2017). Monocarboxylate transporter MCT1 promotes tumor metastasis independently of its activity as a lactate transporter. Cancer Res.

[33] Martin TA, Jiang WG (2009). Loss of tight junction barrier function and its role in cancer metastasis. Biochim Biophys Acta.

[34] Long X, Wong CC, Tong L, Chu ESH, Ho Szeto C, Go MYY (2019). *Peptostreptococcus anaerobius* promotes colorectal carcinogenesis and modulates tumour immunity. Nat Microbiol.

[35] Chung L, Thiele Orberg E, Geis AL, Chan JL, Fu K, DeStefano Shields CE (2018). *Bacteroides fragilis* toxin coordinates a pro-carcinogenic inflammatory cascade via targeting of colonic epithelial cells. Cell Host Microbe.

[36] Cuevas-Ramos G, Petit CR, Marcq I, Boury M, Oswald E, Nougayrède JP (2010). Escherichia coli induces DNA damage in vivo and triggers genomic instability in mammalian cells. Proc Natl Acad Sci USA.

[37] Nowrouzian FL, Oswald E (2012). *Escherichia coli* strains with the capacity for long-term persistence in the bowel microbiota carry the potentially genotoxic pks island. Microb Pathog.

[38] Secher T, Samba-Louaka A, Oswald E, Nougayrède JP (2013). *Escherichia coli* producing colibactin triggers premature and transmissible senescence in mammalian cells. PLoS ONE.

[39] Wu S, Rhee KJ, Zhang M, Franco A, Sears CL (2007). Bacteroides fragilis toxin stimulates intestinal epithelial cell shedding and gamma-secretase-dependent E-cadherin cleavage. J Cell Sci.

[40] Feng Q, Liang S, Jia H, Stadlmayr A, Tang L, Lan Z (2015). Gut microbiome development along the colorectal adenoma-carcinoma sequence. Nat Commun.

[41] Dai Z, Coker OO, Nakatsu G, Wu WKK, Zhao L, Chen Z (2018). Multi-cohort analysis of colorectal cancer metagenome identified altered bacteria across populations and universal bacterial markers. Microbiome..

[42] Castellarin M, Warren RL, Freeman JD, Dreolini L, Krzywinski M, Strauss J (2012). *Fusobacterium nucleatum* infection is prevalent in human colorectal carcinoma. Genome Res.

[43] Kostic AD, Gevers D, Pedamallu CS, Michaud M, Duke F, Earl AM (2012). Genomic analysis identifies association of Fusobacterium with colorectal carcinoma. Genome Res.

[44] Bullman S, Pedamallu CS, Sicinska E, Clancy TE, Zhang X, Cai D (2017). Analysis of Fusobacterium persistence and antibiotic response in colorectal cancer. Science.

[45] Rubinstein MR, Baik JE, Lagana SM, Han RP, Raab WJ, Sahoo D, et al. *Fusobacterium nucleatum* promotes colorectal cancer by inducing Wnt/β-catenin modulator Annexin A1. EMBO Rep. 2019;20:e47638. 10.15252/embr.201847638 PMC6446206 30833345

[46] Brennan CA, Garrett WS (2019). *Fusobacterium nucleatum*—symbiont, opportunist and oncobacterium. Nat Rev Microbiol.

[47] Mima K, Nishihara R, Qian ZR, Cao Y, Sukawa Y, Nowak JA (2016). *Fusobacterium nucleatum* in colorectal carcinoma tissue and patient prognosis. Gut..

[48] Scott AJ, Alexander JL, Merrifield CA, Cunningham D, Jobin C, Brown R (2019). International Cancer Microbiome Consortium consensus statement on the role of the human microbiome in carcinogenesis. Gut..

[49] Ascierto PA, Long GV, Robert C, Brady B, Dutriaux C, Di Giacomo AM (2019). Survival outcomes in patients with previously untreated BRAF wild-type advanced melanoma treated with nivolumab therapy: three-year follow-up of a Randomized Phase 3 Trial. JAMA Oncol.

[50] Larkin J, Chiarion-Sileni V, Gonzalez R, Grob JJ, Rutkowski P, Lao CD (2019). Five-year survival with combined nivolumab and ipilimumab in advanced melanoma. N Engl J Med.

[51] Ubeda C, Djukovic A, Isaac S (2017). Roles of the intestinal microbiota in pathogen protection. Clin Transl Immunology.

[52] Schreiber RD, Old LJ, Smyth MJ (2011). Cancer immunoediting: integrating immunity’s roles in cancer suppression and promotion. Science.

[53] Ravi R, Noonan KA, Pham V, Bedi R, Zhavoronkov A, Ozerov IV (2018). Bifunctional immune checkpoint-targeted antibody-ligand traps that simultaneously disable TGFβ enhance the efficacy of cancer immunotherapy. Nat. Commun..

[54] Ribas A, Wolchok JD (2018). Cancer immunotherapy using checkpoint blockade. Science.

[55] Stower H (2018). The microbiome influence. Nat Med.

[56] Vétizou M, Pitt JM, Daillère R, Lepage P, Waldschmitt N, Flament C (2015). Anticancer immunotherapy by CTLA-4 blockade relies on the gut microbiota. Science.

[57] Sivan A, Corrales L, Hubert N, Williams JB, Aquino-Michaels K, Earley ZM (2015). Commensal Bifidobacterium promotes antitumor immunity and facilitates anti-PD-L1 efficacy. Science.

[58] Routy B, Le Chatelier E, Derosa L, Duong CPM, Alou MT, Daillère R (2018). Gut microbiome influences efficacy of PD-1-based immunotherapy against epithelial tumors. Science.

[59] Gopalakrishnan V, Spencer CN, Nezi L, Reuben A, Andrews MC, Karpinets TV (2018). Gut microbiome modulates response to anti-PD-1 immunotherapy in melanoma patients. Science.

[60] Matson V, Fessler J, Bao R, Chongsuwat T, Zha Y, Alegre ML (2018). The commensal microbiome is associated with anti-PD-1 efficacy in metastatic melanoma patients. Science.

[61] Gharaibeh RZ, Jobin C (2019). Microbiota and cancer immunotherapy: in search of microbial signals. Gut..

[62] Frankel AE, Coughlin LA, Kim J, Froehlich TW, Xie Y, Frenkel EP (2017). Metagenomic shotgun sequencing and unbiased metabolomic profiling identify specific human gut microbiota and metabolites associated with immune checkpoint therapy efficacy in melanoma patients. Neoplasia..

[63] Limeta A, Ji B, Levin M, Gatto F, Nielsen J (2020). Meta-analysis of the gut microbiota in predicting response to cancer immunotherapy in metastatic melanoma. JCI Insight.

[64] Routy B, Gopalakrishnan V, Daillere R, Zitvogel L, Wargo JA, Kroemer G (2018). The gut microbiota influences anticancer immunosurveillance and general health. Nat Rev Clin Oncol.

[65] Peters BA, Wilson M, Moran U, Pavlick A, Izsak A, Wechter T (2019). Relating the gut metagenome and metatranscriptome to immunotherapy responses in melanoma patients. Genome Med.

[66] Wind TT, Gacesa R, Vich Vila A, de Haan JJ, Jalving M, Weersma RK (2020). Gut microbial species and metabolic pathways associated with response to treatment with immune checkpoint inhibitors in metastatic melanoma. Melanoma Res.

[67] Larkin J, Hodi FS, Wolchok JD (2015). Combined nivolumab and ipilimumab or monotherapy in untreated melanoma. N Engl J Med.

[68] Chaput N, Lepage P, Coutzac C, Soularue E, Le Roux K, Monot C, et al. Baseline gut microbiota predicts clinical response and colitis in metastatic melanoma patients treated with ipilimumab. Ann Oncol. 2017;28:1368–79. 10.1093/annonc/mdx108 28368458

[69] Feng Q, Liang S, Jia H, Stadlmayr A, Tang L, Lan Z (2015). Gut microbiome development along the colorectal adenoma-carcinoma sequence. Nat Commun.

[70] Mager LF, Burkhard R, Pett N, Cooke NCA, Brown K, Ramay H (2020). Microbiome-derived inosine modulates response to checkpoint inhibitor immunotherapy. Science.

[71] Wang T, Gnanaprakasam JNR, Chen X, Kang S, Xu X, Sun H (2020). Inosine is an alternative carbon source for CD8+-T-cell function under glucose restriction. Nat Metab.

[72] Biagi E, Zama D, Nastasi C, Consolandi C, Fiori J, Rampelli S (2015). Gut microbiota trajectory in pediatric patients undergoing hematopoietic SCT. Bone Marrow Transplant.

[73] Holler E, Butzhammer P, Schmid K, Hundsrucker C, Koestler J, Peter K (2014). Metagenomic analysis of the stool microbiome in patients receiving allogeneic stem cell transplantation: loss of diversity is associated with use of systemic antibiotics and more pronounced in gastrointestinal graft-versus-host disease. Biol Blood Marrow Transplant.

[74] Jenq RR, Ubeda C, Taur Y, Menezes CC, Khanin R, Dudakov JA (2012). Regulation of intestinal inflammation by microbiota following allogeneic bone marrow transplantation. J Exp Med.

[75] Peled JU, Devlin SM, Staffas A, Lumish M, Khanin R, Littmann ER (2017). Intestinal microbiota and relapse after hematopoietic-cell transplantation. J. Clin Oncol.

[76] Zama D, Bossù G, Leardini D, Muratore E, Biagi E, Prete A (2020). Insights into the role of intestinal microbiota in hematopoietic stem-cell transplantation. Ther Adv Hematol.

[77] Yu J, Sun H, Cao W, Han L, Song Y, Wan D (2020). Applications of gut microbiota in patients with hematopoietic stem-cell transplantation. Exp Hematol Oncol..

[78] Iida N, Dzutsev A, Stewart CA, Smith L, Bouladoux N, Weingarten RA (2013). Commensal bacteria control cancer response to therapy by modulating the tumor microenvironment. Science.

[79] Gui QF, Lu HF, Zhang CX, Xu ZR, Yang YH (2015). Well-balanced commensal microbiota contributes to anti-cancer response in a lung cancer mouse model. Genet Mol Res.

[80] Daillère R, Vétizou M, Waldschmitt N, Yamazaki T, Isnard C, Poirier-Colame V (2016). *Enterococcus hirae* and *Barnesiella intestinihomini*s facilitate cyclophosphamide-induced therapeutic immunomodulatory effects. Immunity..

[81] Jordan KR, Loman BR, Bailey MT, Pyter LM (2018). Gut microbiota-immune-brain interactions in chemotherapy-associated behavioral comorbidities. Cancer..

[82] Bell JS, Spencer JI, Yates RL, Yee SA, Jacobs BM, DeLuca GC (2019). Invited review: from nose to gut—the role of the microbiome in neurological disease. Neuropathol Appl Neurobiol.

[83] Ramakrishna C, Corleto J, Ruegger PM, Logan GD, Peacock BB, Mendonca S (2019). Dominant role of the gut microbiota in chemotherapy induced neuropathic pain. Scientific Rep.

[84] Elkrief A, Derosa L, Kroemer G, Zitvogel L, Routy B (2019). The negative impact of antibiotics on outcomes in cancer patients treated with immunotherapy: a new independent prognostic factor?. Ann Oncol.

[85] Derosa L, Routy B, Fidelle M, Iebba V, Alla L, Pasolli E (2020). Gut bacteria composition drives primary resistance to cancer immunotherapy in renal cell carcinoma patients. Eur Urol.

[86] Hakozaki T, Richard C, Elkrief A, Hosomi Y, Benlaïfaoui M, Mimpen I (2020). The Gut microbiome associates with immune checkpoint inhibition outcomes in patients with advanced non-small cell lung cancer. Cancer Immunol Res..

[87] Manichanh C, Reeder J, Gibert P, Varela E, Llopis M, Antolin M (2010). Reshaping the gut microbiome with bacterial transplantation and antibiotic intake. Genome Res.

[88] Raymond F, Deraspe M, Boissinot M, Bergeron MG, Corbeil J (2016). Partial recovery of microbiomes after antibiotic treatment. Gut Microbes.

[89] Derosa L, Hellmann MD, Spaziano M, Halpenny D, Fidelle M, Rizvi H (2018). Negative association of antibiotics on clinical activity of immune checkpoint inhibitors in patients with advanced renal cell and non-small-cell lung cancer. Ann Oncol.

[90] Pinato DJ, Howlett S, Ottaviani D, Urus H, Patel A, Mineo T, et al. Association of prior antibiotic treatment with survival and response to immune checkpoint inhibitor therapy in patients with cancer. JAMA Oncol. 2019;5:1774–8. 10.1001/jamaoncol.2019.2785 PMC6743060 31513236

[91] Cheung KS, Leung WK (2019). Long-term use of proton-pump inhibitors and risk of gastric cancer: a review of the current evidence. Ther Adv Gastroenterol.

[92] Jackson MA, Verdi S, Maxan M-E, Shin CM, Zierer J, Bowyer RCE (2018). Gut microbiota associations with common diseases and prescription medications in a population-based cohort. Nat Commun.

[93] Imhann F, Bonder MJ, Vich Vila A, Fu J, Mujagic Z, Vork L (2016). Proton pump inhibitors affect the gut microbiome. Gut..

[94] Cheung KS, Chan EW, Wong AYS, Chen L, Wong ICK, Leung WK (2018). Long-term proton pump inhibitors and risk of gastric cancer development after treatment for <em>Helicobacter pylori</em>: a population-based study. Gut..

[95] Homicsko K, Richtig G, Tuchmann F, Tsourti Z, Hanahan D, Coukos G, et al. Proton pump inhibitors negatively impact survival of PD-1 inhibitor based therapies in metastatic melanoma patients. England: Oxford Univ. Press; 2018. p. 40.

[96] Arbour KC, Mezquita L, Long N, Rizvi H, Auclin E, Ni A (2018). Impact of baseline steroids on efficacy of programmed cell death-1 and programmed death-ligand 1 blockade in patients with non-small-cell lung cancer. J Clin Oncol.

[97] Huang EY, Inoue T, Leone VA, Dalal S, Touw K, Wang Y (2015). Using corticosteroids to reshape the gut microbiome: implications for inflammatory bowel diseases. Inflamm Bowel Dis.

[98] Rohlke F, Stollman N (2012). Fecal microbiota transplantation in relapsing *Clostridium difficile* infection. Ther Adv Gastroenterol.

[99] van Nood E, Vrieze A, Nieuwdorp M, Fuentes S, Zoetendal EG, de Vos WM (2013). Duodenal infusion of donor feces for recurrent *Clostridium difficile*. N Engl J Med.

[100] Kakihana K, Fujioka Y, Suda W, Najima Y, Kuwata G, Sasajima S (2016). Fecal microbiota transplantation for patients with steroid-resistant acute graft-versus-host disease of the gut. Blood..

[101] Bel S, Elkis Y, Elifantz H, Koren O, Ben-Hamo R, Lerer-Goldshtein T (2014). Reprogrammed and transmissible intestinal microbiota confer diminished susceptibility to induced colitis in TMF-/- mice. Proc Natl Acad Sci USA.

[102] Baruch EN, Youngster I, Ben-Betzalel G, Ortenberg R, Lahat A, Katz L (2021). Fecal microbiota transplant promotes response in immunotherapy-refractory melanoma patients. Science.

[103] Wang Y, Wiesnoski DH, Helmink BA, Gopalakrishnan V, Choi K, DuPont HL (2018). Fecal microbiota transplantation for refractory immune checkpoint inhibitor-associated colitis. Nat Med.

[104] Fasanello MK, Robillard KT, Boland PM, Bain AJ, Kanehira K (2020). Use of fecal microbial transplantation for immune checkpoint inhibitor Colitis. ACG Case Rep J.

[105] O’Toole PW, Marchesi JR, Hill C (2017). Next-generation probiotics: the spectrum from probiotics to live biotherapeutics. Nat Microbiol.

[106] Khalesi S, Bellissimo N, Vandelanotte C, Williams S, Stanley D, Irwin C (2019). A review of probiotic supplementation in healthy adults: helpful or hype?. Eur J Clin Nutr.

[107] Suez J, Zmora N, Zilberman-Schapira G, Mor U, Dori-Bachash M, Bashiardes S (2018). Post-antibiotic gut mucosal microbiome reconstitution is impaired by probiotics and improved by autologous FMT. Cell..

[108] Spencer CN, Gopalakrishnan V, McQuade J, Andrews M, Helmink B, Wadud Khan M, et al. The gut microbiome of melanoma patients is distinct from healthy controls, and associations with treatment outcomes are influenced by host lifestyle factors. Presented at AACR Annual Meeting 2019 Media Preview; 2019 Feb 27; Philadelphia, Pennsylvania.

[109] Gopalakrishnan V, Spencer CN, McQuade JL, Andrews M, Helmink B, Cogdill AP, et al. The gut microbiome of metastatic melanoma patients initiating systemic therapy is influenced by host factors including diet, probiotic and antibiotic use [abstract]. In: Annual Meeting of the Society for Immunotherapy of Cancer SITC 2018; 2018 Nov 7–11; Washington, DC. Abstract No. P505.

[110] Redman MG, Ward EJ, Phillips RS (2014). The efficacy and safety of probiotics in people with cancer: a systematic review. Ann Oncol.

[111] Mego M, Holec V, Drgona L, Hainova K, Ciernikova S, Zajac V (2013). Probiotic bacteria in cancer patients undergoing chemotherapy and radiation therapy. Complement Ther Med.

[112] Lalla RV, Bowen J, Barasch A, Elting L, Epstein J, Keefe DM (2014). MASCC/ISOO clinical practice guidelines for the management of mucositis secondary to cancer therapy. Cancer..

[113] Kuugbee ED, Shang X, Gamallat Y, Bamba D, Awadasseid A, Suliman MA (2016). Structural change in microbiota by a probiotic cocktail enhances the gut barrier and reduces cancer via TLR2 signaling in a rat model of colon cancer. Dig Dis Sci.

[114] Ogawa T, Asai Y, Tamai R, Makimura Y, Sakamoto H, Hashikawa S (2006). Natural killer cell activities of synbiotic *Lactobacillus casei* ssp. *casei* in conjunction with dextran. Clin Exp Immunol.

[115] Rafter J, Bennett M, Caderni G, Clune Y, Hughes R, Karlsson PC (2007). Dietary synbiotics reduce cancer risk factors in polypectomized and colon cancer patients. Am J Clin Nutr.

[116] Hays SG, Patrick WG, Ziesack M, Oxman N, Silver PA (2015). Better together: engineering and application of microbial symbioses. Curr Opin Biotechnol.

[117] Tanoue T, Morita S, Plichta DR, Skelly AN, Suda W, Sugiura Y (2019). A defined commensal consortium elicits CD8 T cells and anti-cancer immunity. Nature..

[118] David LA, Maurice CF, Carmody RN, Gootenberg DB, Button JE, Wolfe BE (2014). Diet rapidly and reproducibly alters the human gut microbiome. Nature..

[119] Reynolds A, Mann J, Cummings J, Winter N, Mete E, Te Morenga L (2019). Carbohydrate quality and human health: a series of systematic reviews and meta-analyses. Lancet.

[120] Woo JK, Choi S, Kang JH, Kim DE, Hurh BS, Jeon JE (2016). Fermented barley and soybean (BS) mixture enhances intestinal barrier function in dextran sulfate sodium (DSS)-induced colitis mouse model. BMC Complement Altern Med.

[121] McDonald D, Hyde E, Debelius JW, Morton JT, Gonzalez A, Ackermann G (2018). American Gut: an open platform for citizen science microbiome research. mSystems.

[122] Vinson JA, Su X, Zubik L, Bose P (2001). Phenol antioxidant quantity and quality in foods: fruits. J Agric Food Chem.

[123] Tzounis X, Rodriguez-Mateos A, Vulevic J, Gibson GR, Kwik-Uribe C, Spencer JP (2011). Prebiotic evaluation of cocoa-derived flavanols in healthy humans by using a randomized, controlled, double-blind, crossover intervention study. Am J Clin Nutr.

[124] Zhang K, Dai H, Liang W, Zhang L, Deng Z (2019). Fermented dairy foods intake and risk of cancer. Int J Cancer.

[125] Zang J, Shen M, Du S, Chen T, Zou S (2015). The association between dairy intake and breast cancer in western and asian populations: a systematic review and meta-analysis. J Breast Cancer.

[126] Nettleton JE, Reimer RA, Shearer J (2016). Reshaping the gut microbiota: impact of low calorie sweeteners and the link to insulin resistance?. Physiol Behav.

[127] Bian X, Chi L, Gao B, Tu P, Ru H, Lu K (2017). Gut microbiome response to sucralose and its potential role in inducing liver inflammation in mice. Front Physiol.

[128] Chassaing B, Koren O, Goodrich JK, Poole AC, Srinivasan S, Ley RE (2015). Dietary emulsifiers impact the mouse gut microbiota promoting colitis and metabolic syndrome. Nature..

[129] Aykan NF (2015). Red meat and colorectal cancer. Oncol Rev.

[130] Russell WR, Gratz SW, Duncan SH, Holtrop G, Ince J, Scobbie L (2011). High-protein, reduced-carbohydrate weight-loss diets promote metabolite profiles likely to be detrimental to colonic health. Am J Clin Nutr.

[131] Spector T, Gardner C (2019). Bacon rashers, statistics, and controversy. BMJ.

[132] Lee KA, Shaw HM, Bataille V, Nathan P, Spector TD (2020). Role of the gut microbiome for cancer patients receiving immunotherapy: dietary and treatment implications. Eur J Cancer.

[133] Berry SE, Valdes AM, Drew DA, Asnicar F, Mazidi M, Wolf J (2020). Human postprandial responses to food and potential for precision nutrition. Nat Med.

[134] Chinna Meyyappan A, Forth E, Wallace CJK, Milev R (2020). Effect of fecal microbiota transplant on symptoms of psychiatric disorders: a systematic review. BMC Psychiatry.

